# *Monoraphidium* sp. HDMA-20 is a new potential source of α-linolenic acid and eicosatetraenoic acid

**DOI:** 10.1186/s12944-019-0996-5

**Published:** 2019-03-04

**Authors:** Yimeng Lin, Jingping Ge, Yunye Zhang, Hongzhi Ling, Xiufeng Yan, Wenxiang Ping

**Affiliations:** 1 Key Laboratory of Microbiology, College of Heilongjiang Province, School of Life Sciences, Heilongjiang University, Harbin, People’s Republic of China; 2 Alkali Soil Natural Environmental Science Center, Northeast Forestry University, Harbin, People’s Republic of China; 3 Engineering Research Center of Agricultural Microbiology Technology, Ministry of Education, Heilongjiang University, Harbin, People’s Republic of China

**Keywords:** PUFA, ALA, ETA, Microalgae, *Monoraphidium*

## Abstract

**Background:**

ω-3 polyunsaturated fatty acids (PUFAs) are synthesized from α-Linolenic acid (ALA, C18:3ω3) and play important roles in anti-inflammatory and antioxidant responses in mammal cells. ALA is an essential fatty acid which cannot be produced within the human body and must be acquired through diet. The purpose of this study was to evaluate the potential of a novel microalgal strain (HDMA-20) as a source of ω-3 PUFAs including ALA and eicosatetraenoic acid (ETA, C20:4ω3).

**Method:**

Phylogenetic Neighbor-Joining analysis based on 18S ribosomal DNA sequence was used to identify the microalga strain HDMA-20. Autotrophic condition was chosen to cultivate HDMA-20 to reduce the cultivation cost. GC-MS was used to determine the fatty acid composition of HDMA-20 lipid.

**Results:**

A microalgal strain (HDMA-20) from Lake Chengfeng (Daqing, Heilongjiang province, China) was found to accumulate high content of ω-3 PUFAs (63.4% of total lipid), with ALA and eicosatetraenoic acid (ETA, C20:4ω3) accounting for 35.4 and 9.6% of total lipid, respectively. Phylogenetic analysis based on 18S ribosomal DNA sequences suggested that the HDMA-20 belonged to genus *Monoraphidium* (Selenastraceae, Sphaeropleales) and its 18S rDNA sequence information turned out to be new molecular record of *Monoraphidium* species. The biomass productivity and lipid content of HDMA-20 were also investigated under autotrophic condition. The biomass productivity of HDMA-20 reached 36.3 mg L^− 1^ day^− 1^, and the lipid contents was 22.6% of dry weight.

**Conclusion:**

HDMA-20 not only represent an additional source of ALA, but also a totally new source of ETA. The high content of ω-3 PUFAs, especially ALA, of HDMA-20, makes it suitable as a source of nutrition supplements for human health. In addition, HDMA-20 exhibited good properties in growth and lipid accumulation, implying its potential for cost-effective ω-3 PUFAs production in future.

## Background

Polyunsaturated fatty acids (PUFAs) have two main classes of fatty acids, omega-6 (ω-6) and omega-3 (ω-3) PUFAs. Both of them are important components of the human diet and play significant roles in cell signaling, membrane structure and function, and many other physiological responses. Intake of ω-3 PUFA has been shown to ameliorate inflammatory and cardiovascular diseases [1, 2]. In contrast, high ω-6 PUFA intake has been associated with inflammatory responses, cardiovascular diseases, Alzheimer’s disease, etc. [3]. Therefore, a lower ratio of ω-6/ω-3 PUFAs is recommended in order to reduce the risk of many chronic diseases. Unfortunately, ω-3 PUFAs are generally lacking in the diet of industrialized societies, whereas excessive amounts ω-6 PUFAs are often consumed [4]. As a result, increased intake of ω-3 fatty acids are highly suggested to keep a balanced ratio of ω-6 to ω-3 PUFAs in the human diet.

α-Linolenic acid (ALA, C18:3ω3) is an octadecatrienoic acid with three cis double bonds at the 9, 12 and 15 positions. As one of ω-3 fatty acids, ALA has been reported to have nutraceutical/pharmacological benefits and is safe as a food ingredient [5]. ALA exhibits a variety of health benefits, such as endogenous neurorestoration [6], reducing the risk of nonfatal acute myocardial infarction and coronary heart disease [7, 8], and anti-cancer effect [9].

In mammalian cells ALA is the substrate of a series of elongation and desaturation reactions to generate long chain ω-3 PUFAs. Eicosatetraenoic acid (ETA, C20:4ω3) is an intermediate metabolite in the ω-3 pathway. With many research focused on the health benefit of ALA, the biochemical function of ETA has been little investigated. Early studies showed that ETA can modulate eicosanoid production in mammalian cell systems and works as active molecule responsible for the anti-inflammatory responses [10, 11]. Recently, it is suggested that low levels of ETA and high levels of vaccenic acid (C18:1ω7) were significantly associated with disease severity and mortality in the chronic heart failure [12]. The research on ETA is relatively limited, partly because the difficulty of obtaining ETA in high purity since the low content of ETA in natural oils. The major natural source of ETA is fish oil, which have only about 1–2% ETA of total fatty acids [11]. The natural source of ETA is remaining to be explored.

In the downstream of ω-3 fatty acids metabolic pathway, another two important ω-3 PUFA, EPA (cis-5, 8, 11, 14, 17-eicosapentaenoic acid, C20:5ω3) and DHA (cis-4, 7, 10, 13, 16, 19-docosahexaenoic acid, C22:6ω3), were synthesized. EPA and DHA are major components of brain cells and crucial for the cardiovascular health, the nervous system functioning, etc. [5]. The traditional source of EPA and DHA is fish oil, which is a limited resource and much susceptible to contaminations. Compared with EPA and DHA, ALA exhibited comparable cardiovascular disease benefits, which makes it an important alternative source of EPA and DHA [13].

As the precursor of the ω-3 PUFA metabolic pathway, ALA is extremely important for the sufficient supply of long chain ω-3 fatty acids. Because mammals cannot synthesize ALA de novo, ALA must be included in the human diet, which is why ALA is called essential fatty acid. ALA is primarily found in plant source. The richest amounts of ALA from plant sources are flaxseed and camelina oils, which have 53 and 38% (in average) of ALA respectively. Unfortunately, flaxseed oil is consumed by only a limited population, whereas camelina oil is not commonly consumed as part of the diet [5]. Vegetable oils are the common dietary source of ALA, but the ALA contents are relatively low. As examples, rapeseed and peanut oil contain 8.1 and 0.5% of ALA respectively [14]. Recently, microalgae as potential sources of ω-3 PUFAs have raised a great interest. Microalgae are photosynthetic microbes which have been considered as promising sources of high-value compounds such as pigments, sugars and lipids [15]. Compared with conventional oil crops, microalgae as lipid producer present quite a few advantages, such as rapid growth rates, high oil content, not requiring arable land. ω-3 PUFAs including DHA, EPA and ALA are abundant in some particular microalgal strains, which can be used as dietary supplement to improve nutritional status of animal feed, further producing ω-3 PUFA enriched food for human health [16, 17].

In this work, a novel microalgal strain HDMA-20 was found to accumulate high content of ω-3 PUFAs, with a considerable amount of ALA and ETA. The lipid produced by HDMA-20 presented a promising ω-6/ω-3 ratio of 1/30.7, which is much less than the alert value (10/1) suggested by the World Health Organization (WHO), implying that HDMA-20 may serve as a favorable source of health promoting compounds to improve the nutritional status caused by ω-3 PUFAs deficiency in modern diet.

## Methods

### Isolation and growth condition

Microalgal strain HDMA-20 was isolated from water samples collected from Lake Chengfeng (46°53′ N and 124°89′ E), which located in Daqing, a city in the west of Heilongjiang province (China). Water samples were filtered through six layers of gauze to remove protozoa, and then used as an inoculum. 10 mL of filtered sample was added into 250-mL flask containing 100 mL of liquid BG-11 medium. The culture was kept at 28 °C for about 20 days and plated onto BG-11 solid medium, which was cultivated at 28 °C until single colonies appeared. Microalgae were purified by picking up colonies and serial streak plating, followed by microscopic examination, until individual pure colonies were isolated.

For autotrophic culture, a 200-mL BG-11 medium in a 500-mL flask was inoculated with about 40 mL of pure microalgal strains, to allow the optical density at 680 nm to reach 0.3. Isolates were cultured under a 16 h light and 8 h dark cycle at 28 °C and shaken by hand three times per day to prevent the algae cells from settling on the surface of the flask. Cell counting in a haemocytometer was conducted every other day using an Olympus optical microscope to develop the growth curves.

### Molecular identification

Total genomic DNA was extracted from 50 mg dried algae biomass using the Biospin Fungus Genomic DNA Extraction Kit (Bioer Technology, Hangzhou, China), following the manufacturer’s instruction. Two primers, namely, 18S-F (5′-ACCTGGTTGATCCTGCCAGT-3′), 18S-R (5′-TCACCTACGGAAACCTTG T-3′), were used in PCR reactions. PCR was performed in 25 μL reaction system containing 2 × PrimeSTAR Max Premix (Takara, Japan), 0.4 μM of each primer, 50 ng genomic DNA. The program of PCR amplification is as follows: an initial preheating of 5 min at 94 °C, 35 cycles of denaturation at 98 °C for 10 s, annealing at 55 °C for 15 s and extension at 72 °C for 2 min, followed by another 8 min final extension at 72 °C. The amplified PCR products were examined by electrophoresis and sequenced by the Sangon Biotech Co., Ltd. (Shanghai, China).

### Phylogenetic analysis of the isolates

The 18S ribosomal DNA sequences of HDMA-20 was used as query sequence to search for publicly available sequences in NCBI Genbank database using the basic local alignment search tool (BLAST). 15 sequences having the highest similarity to each query sequence were obtained and used for phylogenetic analyses (accession numbers of these sequences are indicated after the name of each strain). Multiple sequence alignment was carried out using the ClustalW [18]. The Neighbor-Joining algorithm was used to construct phylogenetic tree in the software Molecular Genetics Analysis (MEGA) 6.06 [19]. Bootstrap tests values based on 1000 re-samplings of the sequences were chosen to determine the statistical reliability of the tree’s topology. *Chlamydomonas* sp. GTD3a-5 (KC149966) was used as an outgroup to define the root of trees.

### Fatty acid profile analysis

Approximately 1 g of lyophilized biomass was ground and saponified with 0.4 M KOH solution in methanol at 70 °C for 10 min. Fatty acids in the mixture were methylated using a BF_3_-methanol solution at 70 °C for another 10 min after the mixture was cooled to room temperature. After methylation, 5 mL of n-hexane was added into the mixture, the top layer was then separated and placed into a phial for gas chromatography/mass spectrometry (GC/MS) analysis.

GC/MS analysis was carried out in an Agilent 7890A/5975C system (Agilent Technologies, Santa Clara, CA), which was equipped with an HP-5MS capillary column (30 m × 0.25 mm × 0.25 μm). The injector temperature was kept at 250 °C, with an oven temperature programmed from 50 °C to 170 °C at a rate of 10 °C min^− 1^ after 5 min hold at 50 °C, from 170 °C to 250 °C at a rate of 5 °C min^− 1^ after a 1 min hold time at 170 °C, and then finally maintained isothermally at 250 °C for 15 min. The carrier gas was 99.9% pure helium, with a column flow rate of 0.8 mL min^− 1^. Peaks identification was performed by comparing the mass spectra to the National Institute of Standard and Technology (NIST) mass spectral library (NIST11.L). Experiments were run in triplicate, and data were shown as mean ± standard deviation (SD).

### Biomass estimation and lipid content measurement

The biomass was harvested by centrifugation at 3800 rpm for 20 min after cultivation, washed three times by BG-11 medium, and then dried in an oven at 50 °C for about 12 h. The dried pellet was weighed and considered as dry cell biomass (DCW). Biomass productivity (BP) was calculated using the following equation:

$$ \mathrm{BP}\ \left(\mathrm{mg}\;{\mathrm{L}}^{\hbox{-} 1}\right)=\left({\mathrm{w}}_{\mathrm{b}}-{\mathrm{w}}_{\mathrm{a}}\right)\times {\left({\mathrm{t}}_{\mathrm{b}}-{\mathrm{t}}_{\mathrm{a}}\right)}^{\hbox{-} 1} $$

Where w_a_ and w_b_ were the DCW at the start and the end of the exponential phase respectively, and t_b_ – t_a_ is the cultivation days the exponential phase lasts.

Experiments were performed in triplicate, and data were shown as mean ± standard deviation (SD).

Lipid was extracted using the Folch method [20] with some modifications. The lyophilized biomass was ground into a fine powder. First, about 200 mg of the powder (w_1_) was blended with 6 mL chloroform/methanol (2:1 *v*/v), and the mixture was agitated on a vortex shaker for 10 min at room temperature. Then, the upper phase was recovered from centrifugation at 4000 rpm at 4 °C for 10 min, and the insoluble pellet was re-extracted in 6 mL of chloroform/methanol solution twice. Last, 0.9% Nacl was added into the collected extract and left standing for 15 min so as to separate the solution to two phases. The chloroform phase was evaporated and weighed (w_2_). The lipid content was calculated as percent of dry biomass:

$$ \mathrm{Lipid}\ \mathrm{content}={\mathrm{w}}_2/{\mathrm{w}}_1\times 100\% $$

Where w_1_ is the dry cell biomass and w_2_ is the weight of the total extracted lipids.

The lipid productivity (LP) was calculated using the following equation:

$$ \mathrm{LP}\ \left(\mathrm{mg}\;{\mathrm{L}}^{\hbox{-} 1}{\mathrm{d}}^{\hbox{-} 1}\right)={\mathrm{w}}_2\times {\mathrm{V}}^{\hbox{-} 1}\times {\mathrm{T}}^{\hbox{-} 1} $$

Where V is the volume of the lipid extraction sample, and T is the days of cultivation period.

Moreover, the intracellular lipids of microalgal cells were also visualized via Nile red (Sigma-Aldrich, USA) staining. 3 mL of the cultures was taken and centrifuged at 3800 rpm for 20 min, and the pellet was re-suspended in 0.5 mL phosphate buffer saline solution (0.1 M) at room temperature. The pellet was then re-suspended in 1.5 mL DMSO solution and vortexed for 2 min before adding 30 μL Nile red stain solution (1 mg mL^− 1^) and incubated in the dark for another 5 min at room temperature. Stained cells were visualized under the fluorescent microscope (Olympus, Japan) via UV light with emission and excitation at 575 nm and 530 nm, respectively.

## Results

### Isolation and characterization of HDMA-20

**Fig. 1:**
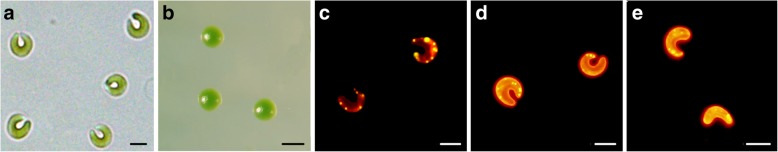
Light microscope image of cells, colonies and lipid accumulation of *Monoraphidium* sp. HDMA-20. Cells and colonies image shown as **a** (scale bar: 5 μm) and **b** (scale bar: 1 mm). The Nile red stained cells with lipid droplets shown in golden color on the 6th day (**c**), 12th day (**d**) and 18th day (**e**) (scale bars: 5 μm)

Microalgae are promising sources for producing high-value compounds. With the growing world population and daily increasing demand of high-quality nutritional supplements, new microalgal strains need to be explored from variously natural environment. So far, information on microalgal strains of lakes in Daqing (China) is scarce. In view of this, water samples were collected from eight Daqing lakes to identify potential microalgal strains. By screening several strains isolated from Daqing lakes, HDMA-20 was chosen for further investigation due to its high lipid content. The identification of HDMA-20 not only contributes to better understanding its characteristics, but also helps us to obtain the existing research background of its related species, which will help to utilize the strain in the future. Microscopic examination was then conducted to identify the strain. Under a light microscope, the cells of HDMA-20 were solitary, longer than broad, and reproductive by serial arrangement of autospores. HDMA-20 cells were arched in semi-circles, gradually tapered toward the apex (Fig. 1a). Cell length ranged from 5 to 9 μm, and cell width ranged from 2 to 3.5 μm. The colonies of HDMA-20 were dark green, nearly spherical, lacking a mucilaginous envelope (Fig. 1b). The feature of HDMA-20 resembles the characterization of genus *Monoraphidium* [21, 22].

*Monoraphidium* is a ubiquitous genus classified under the family Selenastraceae, which includes coccoid green algae with elongated shape. Algae in the Selenastraceae are commonly solitary or colonial, spindle-shaped, straight or curved, and are procreated by autospore formation. Cell dimensions are 5-105 μm in length and 1.5–6.5 μm in width [15]. *Monoraphidium* is distinguished from other genera (*Ankistrodesmus*, *Selenastrum* and *Kirchneriella* etc.) in the family by lacking mucilaginous envelope and their reproduction mode of serially arranged autospores [21]. HDMA-20 was morphologically similar to *Monoraphidium circinale* [22]; however, the former was distinguished by being generally more curved.

The classification of microalgae is not simply defined by their morphology since different species may have similar morpho types. Fawley et al. conducted phylogenetic investigations to evaluate the morphospecies concept in the family Selenastraceae and found that some isolates with similar morphologies actually belong to different lineage, whereas other isolates with highly identical 18S rDNA sequences have very diverse morphologies [23]. For this reason, the total DNAs of HDMA-20 were extracted and used as template for PCR amplification of 18S rDNA sequences.

### PCR amplification and phylogenetic analysis

**Fig. 2:**
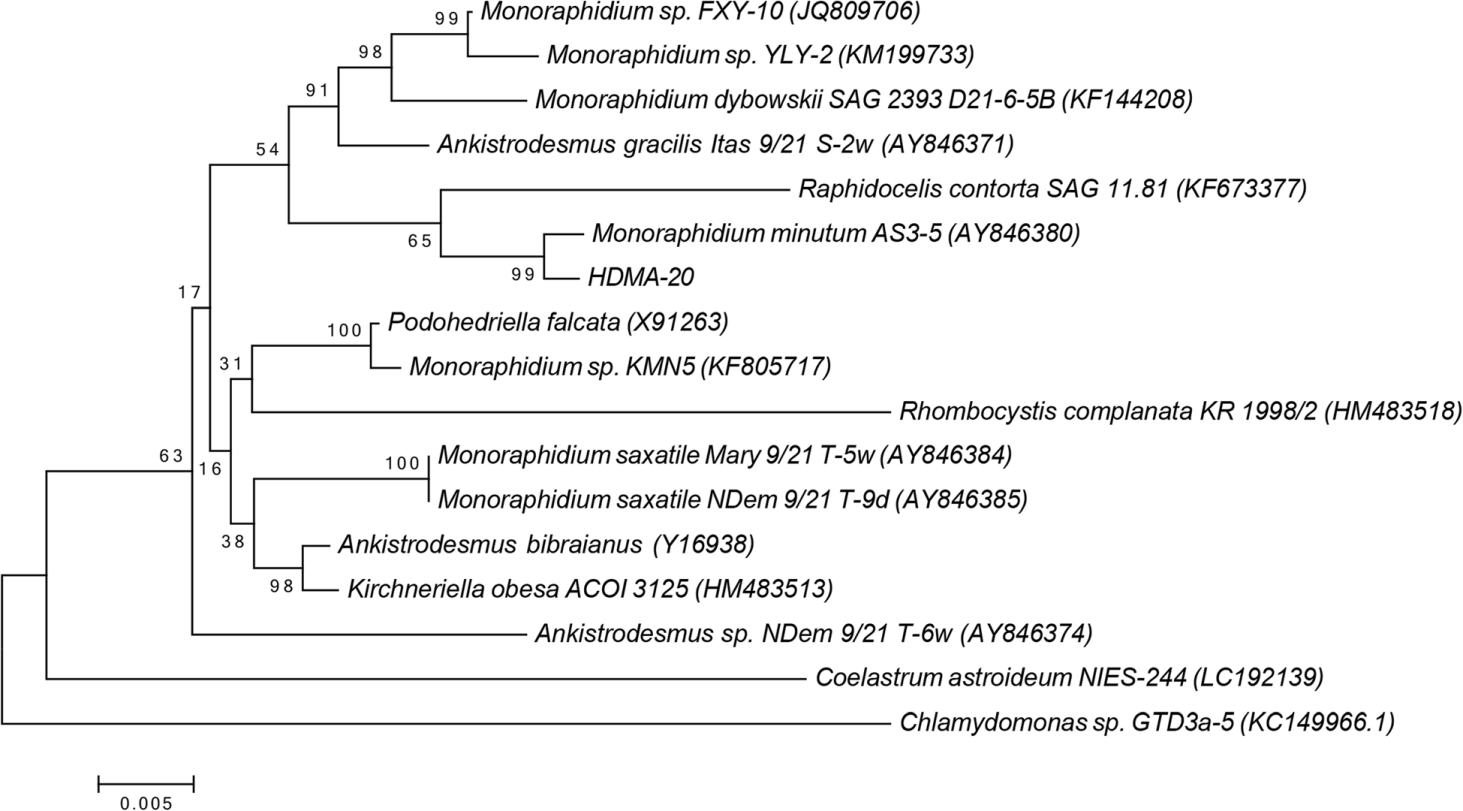
Phylogenetic Neighbor-Joining tree based on 18S rDNA sequence of the isolate HDMA-20. *Chlamydomonas* sp. GTD3a-5 (KC149966) was used as an outgroup to define the root of the tree. The scale bar represents nucleotide substitutions per site

The taxonomy position of HDMA-20 was verified by phylogenetic identification of 18S rDNA gene sequences. PCR reactions were successful in HDMA-20 DNA samples. The phylogenetic characterizations of the HDMA-20 is as follows:

The 18S rDNA sequence of this isolate showed highest similarity (97%) to *Monoraphidium minutum* AS3–5 (AY846380) with an excellent bootstrap support of 99% (Fig. 2b). According to its morphological features, HDMA-20 is close to *M. minutum* [22]; however, this strain is distinguished by having smaller cell sizes and a bigger degree of cell curvature. *Raphidocelis contorta* SAG 11.81 (KF673377) presented 94% identity to HDMA-20 and was clustered in a larger clade with AY846380 and HDMA-20 (Fig. 2b). Aligned with HDMA-20 using ClustalW tool, KF673377 was found to contain a 400 bp gap in its sequence, which may explain why the larger group including KF673377 possessed a much lower support of 65%. It was noteworthy that HDMA-20 and all Selenastraceae (Order Sphaeropleales) strains were grouped in one clade, separated from *Coelastrum astroideum* NIES-244 (LC192139) (Scenedesmaceae, Chlorococcales) and the outgroup (Chlamydomonadaceae, Chlamydomonadales). Hence, it is confirmed that HDMA-20 belongs to the family Selenastraceae. Interestingly, although AY846380 is the most closed member to HDMA-20 in NCBI Genbank, their sequences vary considerably. Compared with AY846380, the sequence of HDMA-20 differs by 2 base insertions, 13 base deletions and 63 base substitutions. However, based on the morphology feature and phylogenetic results, it is most likely HDMA-20 is one member of genus *Monoraphidium*. The strain was thus named as *Monoraphidium* sp. HDMA-20. When the 18S rDNA sequence of HDMA-20 was used as query sequence, there was no identical sequence available on the NCBI DNA database. The sequence information of HDMA-20 tends to be the first molecular record of a novel *Monoraphidium* species.

### Fatty acid composition

The GC-MS analysis of FAMEs produced from *Monoraphidium* sp. HDMA-20 exhibited the following compositions: 27.5% saturated fatty acids (SFAs); 9.1% mono-unsaturated fatty acids (MUFAs); 63.4% polyunsaturated fatty acids (PUFAs). ALA (35.4%), Palmitic acid (22.1%) and Hexadecatetraenoic acid (16.4%) were dominant in the fatty acid composition of *Monoraphidium* sp. HDMA-20. As shown in Table 1, PUFAs were the principal fatty acids in HDMA-20, accounting for over 50 % of fatty acids. Of the total PUFAs, ω-3 PUFA accounted for 96.8%, which reduced the ω-6/ω-3 ratio to a low value of 1/30.7.

**Table 1:** Fatty acid profiles (% of total fatty acids) of *Monoraphidium* sp. HDMA-20 and some edible vegetable oils [14]

Fatty acid	Common name	*Monoraphidium* sp. HDMA-20	Palm	Rapeseed	Sunflower	Corn	Peanut	Mustard
C12:0	Lauric	0.1 ± 0.0						
C14:0	Myristic	0.9 ± 0.0						
C15:0	Pentadecylic^a^	0.5 ± 0.0						
C16:0	Palmitic	23.1 ± 0.5	39.83	3.36	6.6	11.9	8.75	4.32
C16:1ω7	Palmitoleic	8.6 ± 0.2	0.17		0.1			0.21
C16:2ω6	Hexadecadienoic^a^	2.0 ± 0.1						
C16:4ω3	Hexadecatetraenoic^a^	16.4 ± 0.6						
C17:1ω7	Heptadecenoic^a^	0.5 ± 0.0						
C18:0	Stearic	1.0 ± 0.0	5.33	1.12	3.08	2	2.14	1.25
C18:1ω9	Oleic	ND	41.9	63.33	17.31	24.9	60.21	9.26
C18:2ω6	Linoleic	ND	11.46	22	73.31	33.65	21.28	13.79
C18:3ω3	Linolenic	35.4 ± 2.1	0.15	8.11			0.54	18.79
C20:0	Arachidic	ND					1.05	0.95
C20:1ω11	Gadoleic	ND					2.16	5.27
C20:2ω6	Eicosadienoic^a^	ND					1.2	0.7
C20:4ω3	Eicosatetraenoic^a^	9.6 ± 2.7						
C22:0	Behenic	1.8 ± 0.1					2.74	1.09
C22:1ω9	Erucic	ND					0.23	40.1
C24:0	Lignoceric	ND					1.64	
ω-6/ω-3		1/30.7	76.4/1	2.7/1	73.3/0	33.7/0	41.6/1	1/1.3
ω-3/PUFA (%)		96.8	0.01	26.9	0	0	2.3	56.5
SFA		27.5 ± 0.6	45.16	4.48	9.68	13.9	16.32	7.61
MUFA		9.1 ± 0.3	42.07	63.33	17.41	24.9	62.6	54.84
PUFA		63.4 ± 5.5	11.61	30.11	73.31	33.65	23.02	33.28

^a^systematic names

ND – below the limit of detection

blank indicates no information available

Data values are means of three replications ± SD. Relative abundances below 1% were not included

**Fig. 3:**
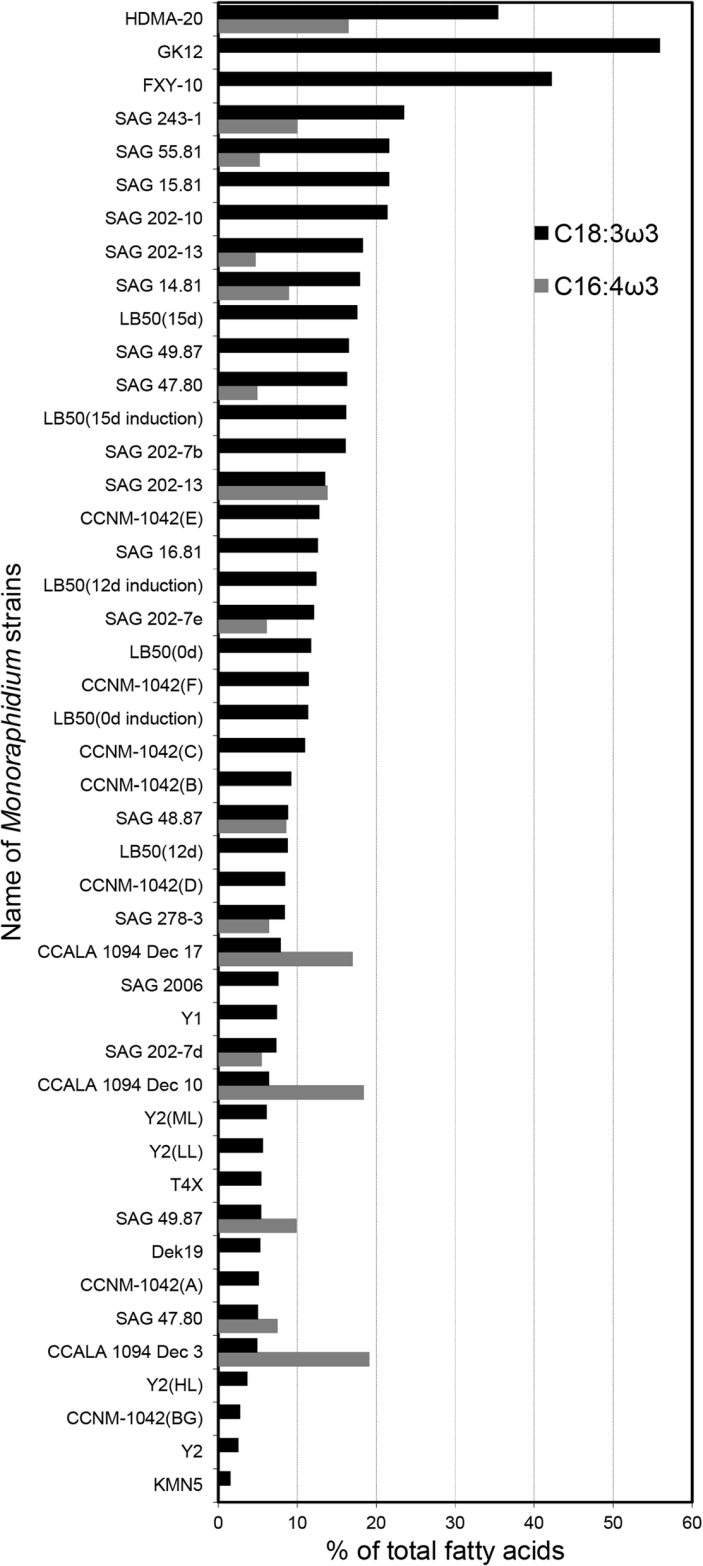
Production of C18:3 ω3 and C16:4 ω3 fatty acids by *Monoraphidium* sp. HDMA-20 and other *Monoraphidium* strains [24–26, 28, 29, 35–38]

In the genus *Monoraphidium*, the PUFAs content ranged from 4.6 to 68.0% of total fatty acids [15, 24, 25]. The PUFA content of HDMA-20 was higher than most of strains in the genus *Monoraphidium*. ALA, as an important ω-3 PUFA, was the most abundant fatty acid in the HDMA-20. The proportion of ALA (35.4%) was higher than all listed vegetable oils (Table 1) and was comparable to that of flaxseed oil (53%) and camelina oil (38%), two known kinds of oils containing the highest ALA content. When compared with other studies of genus *Monoraphidium*, only strain GK12 [26] and FXY-10 [24] were reported to have higher values of ALA than our work (Fig. 3).

All-cis-4,7,10,13-hexadecatetraenoic acid (C16:4ω3) was the second most abundant PUFA, reaching a value of 16.4% in strain HDMA-20. It was found that eicosanoids, which were involved in many allergic and inflammatory processes, were suppressed by C16:4ω3 in MC/9 mouse mast cells [27]. To the best of our knowledge, except for a single study [28], none of other work examining the value of PUFAs in the genus *Monoraphidium* recorded such a high content of C16:4ω3 (Fig. 3).

In the fatty acid composition of HDMA-20, another ω-3 PUFA which constitutes 9.6% of the total fatty acids is ETA (C20:4ω3). In the genus *Monoraphidium*, only one strain *Monoraphidium* sp. GK12 was reported to accumulate ETA, accounting 7.1% of its total fatty acids [26]. The high ETA content (9.6%) makes the strain HDMA-20 unique in the genus *Monoraphidium* and also within the phylum Chlorophyta. According to a comprehensive analysis of fatty acid profiles in more than 2000 microalgal strains, only two strains (*Siderocystopsis punctifera* SAG 28.81 and *Oocystella oogama* SAG 3.96) have higher ETA levels (17.3 and 13.4% respectively) than HDMA-20 within the phylum Chlorophyta [29].

### Culture growth

**Fig. 4:**
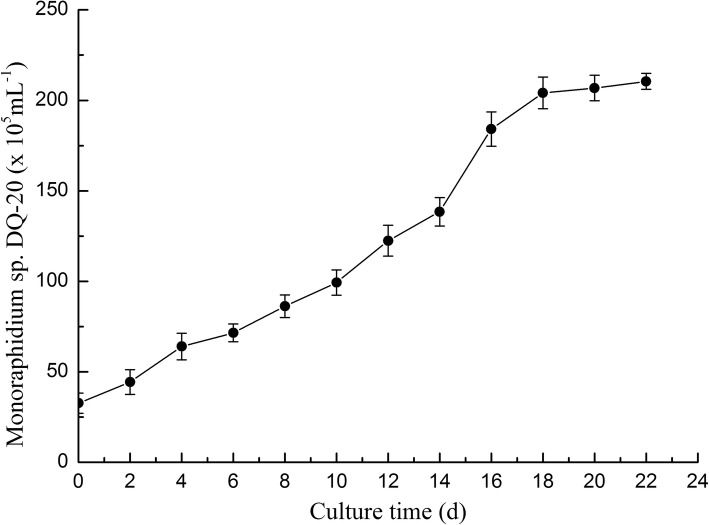
Growth curve of *Monoraphidium* sp. HDMA-20. Cultures were incubated in BG-11 under autotrophic growth mode. Growth was measured by cell numbers counting every other day. Bars are means of three replications ± SD

*Monoraphidium* sp. HDMA-20 can grow in BG-11 medium autotrophically without adding organic carbon source. The growth was measured by counting cell numbers every other day. Data were presented as the means and standard deviations from triplicate determination (Fig. 4). According to the growth curves, no obvious lag phase was observed in *Monoraphidium* sp. HDMA-20. In the phase of cultivation, the growth of cells number was slightly declined after 18 days. For this reason, biomass, biomass productivity, lipid content, and lipid productivity were measured after 18 days even though the strain continued to grow. Table 2 shows that after the cultivation, the biomass of HDMA-20 reached 654 mg L^− 1^. In the genus *Monoraphidium*, the biomass content was reported from 218 to 1518 mg L^− 1^ in the laboratory or small scale under similar photoautotrophic cultivations (Table 2). This put strain HDMA-20 near the average. In consideration of different culturing time each strain possessed, the value of biomass productivity can be more convincing when comparing the biomass value of different strains. As shown in Table 2, the maximum biomass productivity of 36.3 mg L^− 1^ day^− 1^ were obtained by strain HDMA-20. The HDMA-20 cells stained with Nile red were also estimated for the lipid profile under fluorescent microscope (Fig. 1 c, d, e). From the 6th day onward, increased fluorescence was observed in the lipid droplets as the microalgae grew. *Monoraphidium* sp. HDMA-20 accumulated higher lipid on the 18th day. At the same time, the lipid contents of HDMA-20 were 22.6%, while the lipid productivity of these HDMA-20 reached 8.2 mg L^− 1^ day^− 1^. Except for the strain *Monoraphidium* sp. QLY-1 [30], all strains in the genus *Monoraphidium* cited in selected literatures under similar culture conditions showed less values of lipid productivity than HDMA-20.

**Table 2:** Biomass, biomass productivity, lipid content, and lipid productivity of *Monoraphidium* sp. HDMA-20 and other *Monoraphidium* strains [24, 25, 32, 39, 40] under similar photoautotrophic cultivations

*Monoraphidium* strains	Biomass (mg L^−1^)	Biomass productivity (mg L^−1^ day^−1^)	Lipid content (%)	Lipid productivity (mg L^− 1^ day^− 1^)	Media/Scale (L)	References
HDMA-20	654 ± 33	36.3 ± 1.8	22.6 ± 1.9	8.2 ± 0.4	BG-11/0.2	this study
FXY-10	218	12.1	56.8	6.9	BG-11/0.3	[24]
FXY-10	1100	22	44	7	BG-11/0.3	[39]
T4X	200	13	14.3	1.9	BG-11/0.8	[40]
KMN5	650	13.1	34.9	8.2	BBM/0.1	[25]
QLY-1	878 ± 12	21.95 ± 1.31	52.79 ± 1.83	11.59 ± 1.14	BG-11/−	[32]
QLZ-3	1518 ± 3	30.36 ± 1.17	23.80 ± 0.97	7.23 ± 0.51	BG-11/−	[32]
YLY-2	422 ± 18	10.54 ± 1.56	46.55 ± 1.62	4.9 ± 0.37	BG-11/−	[32]

## Discussion

In this study, a novel microalgal strain HDMA-20 was identified as *Monoraphidium* sp. and was tested for its fatty acid composition. The fatty acid profile of HDMA-20 is quite special because its ω-3 fatty acids accounted for the vast majority (96.8%) of total PUFAs. The proportion is much higher than other commonly used vegetable oils (Table 1). In mammalian tissues Linoleic acid (LA, C18:2ω6) and ALA are precursors of ω-6 and ω-3 PUFAs. These PUFAs synthesis reactions are competitive, which means ω-6 and ω-3 pathway are competing for the same desaturases and elongases. In this way, an increased synthesis of PUFAs in one pathway may result in a decrease in the other [31]. Given that ω-6 fatty acid proportion in HDMA-20 is only 2.0% of total lipids (3.2% of PUFAs), it is safe to say that ω-3 PUFA synthetic pathway possessed an overwhelming advantage in HDMA-20. Sunflower oil, however, exhibited a totally different PUFA synthetic characterization, with LA content reaching 73.3% of total lipids and no detectable ω-3 PUFAs (Table 1). There seem to be considerable variations in fatty acid profile between different lipid source. Similar variations of fatty acid profile were also obtained between microalgal species [29]. Therefore, untapped microalgal strains may represent a treasury of tremendous PUFA resources, with great potential to become nutritional supplements for human food. WHO recommended that the ω-6/ω-3 ratio should be lower than 10 in the diet to protect cardiovascular and nervous system and prevent inflammatory responses [32], whereas all the listed vegetable oils except rapeseed and mustard oils offered higher ratio than the recommended value. In this examine, the ω-6/ω-3 ratio of HDMA-20 was 1/30.7, which makes it preferable source for the ω-3 PUFAs intake in human diet.

Another special feature about the fatty acid profile of HDMA-20 is the high content of ALA and ETA, accounting for 35.4 and 9.6% of the total lipids. Considering the important role ALA plays in the cardiovascular health and anti-cancer [7–9], HDMA-20 can serve as a new source of ALA to offer nutraceutical and pharmacological benefits for human health. ETA is a naturally occurring fatty acid which has a role in regulating eicosanoid production and is involved in the anti-inflammatory effects [10, 11]. Unlike its ω-6 series equivalent arachidonic acid (ARA, C20:4ω6), ETA is little investigated due to its rarity in natural oils. Being one of the few ETA sources, marine fish oils have only minor quantities around 1–2% ETA of total fatty acids [11]. High purity ETA is difficult to obtain and extremely expensive, which hampers the investigation on the role of ETA in ω-3 metabolic pathway. Thus, there is a need for alternative sources of ETA in order to meet research demands and nutrition-related requirements. HDMA-20 represent a natural new resource of ETA, which will facilitate the illumination of the ETA role in ω-3 dietary metabolism.

To explore the potential of microalgae as sources of nutrition supplements, a host of efforts can be made to enhance the production economics of microalgal biomass and lipid, such as alga exploring and selection, optimization of culture conditions and downstream processes. Among the measures mentioned above, alga exploring and selection as the first step of the whole process, is believed to be fundamentally vital [33]. An ideal microalgal candidate should grow fast because rapid growth rate means high biomass productivity and less risk of contamination by slowly-growing organisms. It is also expected that microalgal strains accumulate high lipids, which can help increase the lipid productivity and reduce the cost of downstream processes [34]. Furthermore, promising microalgal strains should grow robustly under autotrophic conditions since heterotrophic cultivation requires organic carbon sources, which may increase the cost of raw materials for microalgal production. In this report, a *Monoraphidium* strain HDMA-20 was cultivated under photoautotrophic condition without organic carbon source or extra carbon dioxide. When compared with *Monoraphidium* strains under similar conditions, HDMA-20 achieved good performance in the productivity of biomass and lipid, which makes it potentially promising candidates for producing ω-3 PUFAs. On the other hand, the biomass and lipid productivity of the strain are relatively low, especially when compared with other strains cultivated under heterotrophic conditions. Future work will investigate the optimized combination of factors such as pH, temperature, organic carbon, and the supply of carbon dioxide to further improve the biomass and lipid productivity of HDMA-20.

## Conclusions

In order to identify promising microalgal strains with potential for high-value compounds production, a novel green microalgal strain HDMA-20 was isolated from Lake Chengfeng in Daqing (China). HDMA-20 was identified as *Monoraphidium* sp. based on microscopic examination and genetic characterization. Compared with other strains of this genus under similar autotrophic cultivations, HDMA-20 showed good traits in growth and lipid accumulation. Considering the high content of ω-3 PUFAs and low ω-6/ω-3 ratio, HDMA-20 could be explored further as sources of food supplements to prevent inflammatory-related disorders. Additionally, HDMA-20 as an entirely new source of ETA will help elucidate the function of ETA in ω-3 PUFAs synthetic pathway.

## Abbreviations

ALA: α-Linolenic acid
DHA: cis-4, 7, 10, 13, 16, 19-docosahexaenoic acid
EPA: cis-5, 8, 11, 14, 17-eicosapentaenoic acid
ETA: cis-8, 11, 14, 17-eicosatetraenoic acid
FAME: Fatty acids methyl ester
PCR: Polymerase chain reaction
PUFAs: Polyunsaturated fatty acids

## References

[1] Giudetti AM, Cagnazzo R (2012). Beneficial effects of n-3 PUFA on chronic airway inflammatory diseases. Prostaglandins Other Lipid Mediat..

[2] La Rovere MT, Christensen JH (2015). The autonomic nervous system and cardiovascular disease: role of n-3 PUFAs. Vasc Pharmacol.

[3] Patterson E, Wall R, Fitzgerald GF, Ross RP, Stanton C (2012). Health implications of high dietary omega-6 polyunsaturated fatty acids. J Nutr Metab.

[4] Simopoulos AP (2008). The importance of the omega-6/omega-3 fatty acid ratio in cardiovascular disease and other chronic diseases. Exp Biol Med (Maywood).

[5] Kim KB, Nam YA, Kim HS, Hayes AW, Lee BM (2014). Alpha-linolenic acid: nutraceutical, pharmacological and toxicological evaluation. Food Chem Toxicol.

[6] Piermartiri T, Pan H, Figueiredo TH, Marini AM (2015). Alpha-linolenic acid, a nutraceutical with pleiotropic properties that targets endogenous neuroprotective pathways to protect against organophosphate nerve agent-induced neuropathology. Molecules..

[7] Campos H, Baylin A, Willett WC (2008). Alpha-linolenic acid and risk of nonfatal acute myocardial infarction. Circulation..

[8] Mozaffarian D (2005). Does alpha-linolenic acid intake reduce the risk of coronary heart disease? A review of the evidence. Altern Ther Health Med.

[9] Truan JS, Chen JM, Thompson LU (2010). Flaxseed oil reduces the growth of human breast tumors (MCF-7) at high levels of circulating estrogen. Mol Nutr Food Res.

[10] Croset M, Bordet JC, Lagarde M (1999). Inhibition of prostaglandin H synthase and activation of 12-lipoxygenase by 8,11,14,17-eicosatetraenoic acid in human endothelial cells and platelets. Biochem Pharmacol.

[11] Ghioni C, Porter AEA, Taylor GW, Tocher DR (2002). Metabolism of 18:4n-3 (stearidonic acid) and 20:4n-3 in salmonid cells in culture and inhibition of the production of prostaglandin F 2α (PGF 2α ) from 20:4n-6 (arachidonic acid). Fish Physiol Biochem.

[12] Oie E, Ueland T, Dahl CP, Bohov P, Berge C, Yndestad A, Gullestad L, Aukrust P, Berge RK (2011). Fatty acid composition in chronic heart failure: low circulating levels of eicosatetraenoic acid and high levels of vaccenic acid are associated with disease severity and mortality. J Intern Med.

[13] Fleming JA, Kris-Etherton PM (2014). The evidence for alpha-linolenic acid and cardiovascular disease benefits: comparisons with eicosapentaenoic acid and docosahexaenoic acid. Adv Nutr.

[14] Sajjadi B, Raman AAA, Arandiyan H (2016). A comprehensive review on properties of edible and non-edible vegetable oil-based biodiesel: composition, specifications and prediction models. Renew Sustain Energy Rev.

[15] Yee W (2016). Microalgae from the Selenastraceae as emerging candidates for biodiesel production: a mini review. World J Microbiol Biotechnol.

[16] Stamey JA, Shepherd DM, Veth MJD, Corl BA (2012). Use of algae or algal oil rich in n-3 fatty acids as a feed supplement for dairy cattle. J Dairy Sci.

[17] Toral PG, Hervás G, Gómezcortés P, Frutos P, Juárez M, Fuente MADL (2010). Milk fatty acid profile and dairy sheep performance in response to diet supplementation with sunflower oil plus incremental levels of marine algae. J Dairy Sci.

[18] Thompson JD, Higgins DG, Gibson TJ (1994). CLUSTAL W: improving the sensitivity of progressive multiple sequence alignment through sequence weighting, position-specific gap penalties and weight matrix choice. Nucleic Acids Res.

[19] Tamura K, Stecher G, Peterson D, Filipski A, Kumar S (2013). MEGA6: molecular evolutionary genetics analysis version 6.0. Mol Biol Evol.

[20] Folch J, Lees M, Sloane Stanley GH (1957). A simple method for the isolation and purification of total lipides from animal tissues. J Biol Chem.

[21] Lothar K, Iana U, Thomas F, Huss VAR (2001). Traditional generic concepts versus 18S rRNA gene phylogeny in the green algal family Selenastraceae (Chlorophyceae, Chlorophyta). J Phycol.

[22] Ramos GJP, Bicudo CEM, Neto AG, Moura CEM (2012). Monoraphidium and Ankistrodesmus (Chlorophyceae, Chlorophyta) from Pantanal dos Marimbus, Chapada Diamantina, Bahia state. Brazil Hoehnea.

[23] Fawley MW, Dean ML, Dimmer SK, Fawley KP (2006). Evaluating the morphospecies concept in the Selenastraceae (Chlorophyceae, Chlorophyta). J Phycol.

[24] Yu X, Zhao P, He C, Li J, Tang X, Zhou J, Huang Z (2012). Isolation of a novel strain of Monoraphidium sp. and characterization of its potential application as biodiesel feedstock. Bioresour Technol.

[25] Tale M, Ghosh S, Kapadnis B, Kale S (2014). Isolation and characterization of microalgae for biodiesel production from Nisargruna biogas plant effluent. Bioresour Technol.

[26] Fujii K, Nakashima H, Hashidzume Y, Uchiyama T, Mishiro K, Kadota Y (2010). Potential use of the astaxanthin-producing microalga, Monoraphidium sp. GK12, as a functional aquafeed for prawns. J Appl Phycol.

[27] Ishihara K, Murata M, Kaneniwa M, Saito H, Shinohara K, Maeda-Yamamoto M (1998). Inhibition of icosanoid production in MC/9 mouse mast cells by n-3 polyunsaturated fatty acids isolated from edible marine algae. Biosci Biotechnol Biochem.

[28] Řezanka T, Nedbalová L, Lukavský J, Střížek A, Sigler K (2017). Pilot cultivation of the green alga Monoraphidium sp. producing a high content of polyunsaturated fatty acids in a low-temperature environment. Algal Res.

[29] Lang I, Hodac L, Friedl T, Feussner I. Fatty acid profiles and their distribution patterns in microalgae: a comprehensive analysis of more than 2000 strains from the SAG culture collection. BMC Plant Biol. 2011;11:124–4. 10.1186/1471-2229-11-124 PMC3175173 21896160

[30] Zhao Y, Li D, Ding K, Che R, Xu JW, Zhao P, Li T, Ma H, Yu X (2016). Production of biomass and lipids by the oleaginous microalgae Monoraphidium sp. QLY-1 through heterotrophic cultivation and photo-chemical modulator induction. Bioresour Technol.

[31] Robertson R, Guihéneuf F, Schmid M, Stengel DB, Fitzgerald G, Ross P, Stanton C (2013). Algae-derived polyunsaturated fatty acids: implications for human health.

[32] Sanchezmachado DI, Lopezcervantes J, Lopezhernandez J, Paseirolosada P (2004). Fatty acids, total lipid, protein and ash contents of processed edible seaweeds. Food Chem.

[33] Liu J, Mao X, Zhou W, Guarnieri MT (2016). Simultaneous production of triacylglycerol and high-value carotenoids by the astaxanthin-producing oleaginous green microalga Chlorella zofingiensis. Bioresour Technol.

[34] Sun Z, Zhou ZG, Gerken H, Chen F, Liu J (2015). Screening and characterization of oleaginous Chlorella strains and exploration of photoautotrophic Chlorella protothecoides for oil production. Bioresour Technol.

[35] Yang H, He Q, Hu C (2015). Lipid accumulation by NaCl induction at different growth stages and concentrations in photoautotrophic two-step cultivation of Monoraphidium dybowskii LB50. Bioresour Technol.

[36] Patidar SK, Mitra M, George B, Soundarya R, Mishra S (2014). Potential of Monoraphidium minutum for carbon sequestration and lipid production in response to varying growth mode. Bioresour Technol.

[37] He Q, Yang H, Wu L, Hu C (2015). Effect of light intensity on physiological changes, carbon allocation and neutral lipid accumulation in oleaginous microalgae. Bioresour Technol.

[38] Holbrook GP, Davidson Z, Tatara RA, Ziemer NL, Rosentrater KA, Scott GW (2014). Use of the microalga Monoraphidium sp. grown in wastewater as a feedstock for biodiesel: cultivation and fuel characteristics. Appl Energy.

[39] Che R, Huang L, Yu X (2015). Enhanced biomass production, lipid yield and sedimentation efficiency by iron ion. Bioresour Technol.

[40] Dhup S, Dhawan V (2014). Effect of nitrogen concentration on lipid productivity and fatty acid composition of Monoraphidium sp. Bioresour Technol.

